# eSkip-Finder: a machine learning-based web application and database to identify the optimal sequences of antisense oligonucleotides for exon skipping

**DOI:** 10.1093/nar/gkab442

**Published:** 2021-06-09

**Authors:** Shuntaro Chiba, Kenji Rowel Q Lim, Narin Sheri, Saeed Anwar, Esra Erkut, Md Nur Ahad Shah, Tejal Aslesh, Stanley Woo, Omar Sheikh, Rika Maruyama, Hiroaki Takano, Katsuhiko Kunitake, William Duddy, Yasushi Okuno, Yoshitsugu Aoki, Toshifumi Yokota

**Affiliations:** HPC- and AI-driven Drug Development Platform Division, RIKEN Center for Computational Science, Yokohama 230-0045, Japan; Department of Medical Genetics, University of Alberta Faculty of Medicine and Dentistry, 8613-114 St, Edmonton, AB, Canada; Department of Medical Genetics, University of Alberta Faculty of Medicine and Dentistry, 8613-114 St, Edmonton, AB, Canada; Department of Medical Genetics, University of Alberta Faculty of Medicine and Dentistry, 8613-114 St, Edmonton, AB, Canada; Department of Medical Genetics, University of Alberta Faculty of Medicine and Dentistry, 8613-114 St, Edmonton, AB, Canada; Department of Medical Genetics, University of Alberta Faculty of Medicine and Dentistry, 8613-114 St, Edmonton, AB, Canada; Department of Medical Genetics, University of Alberta Faculty of Medicine and Dentistry, 8613-114 St, Edmonton, AB, Canada; Department of Medical Genetics, University of Alberta Faculty of Medicine and Dentistry, 8613-114 St, Edmonton, AB, Canada; Department of Medical Genetics, University of Alberta Faculty of Medicine and Dentistry, 8613-114 St, Edmonton, AB, Canada; Department of Medical Genetics, University of Alberta Faculty of Medicine and Dentistry, 8613-114 St, Edmonton, AB, Canada; HPC- and AI-driven Drug Development Platform Division, RIKEN Center for Computational Science, Yokohama 230-0045, Japan; Department of Molecular Therapy, National Institute of Neuroscience, National Center of Neurology and Psychiatry (NCNP), Kodaira, Tokyo 187-8551, Japan; Northern Ireland Center for Stratified Medicine, Biomedical Sciences Research Institute, C-TRIC, Altnagelvin Hospital Campus, Ulster University, Londonderry BT47 6SB, UK; HPC- and AI-driven Drug Development Platform Division, RIKEN Center for Computational Science, Yokohama 230-0045, Japan; Department of Biomedical Data Intelligence, Graduate School of Medicine, Kyoto University, Kyoto 606-8507, Japan; Department of Molecular Therapy, National Institute of Neuroscience, National Center of Neurology and Psychiatry (NCNP), Kodaira, Tokyo 187-8551, Japan; Department of Medical Genetics, University of Alberta Faculty of Medicine and Dentistry, 8613-114 St, Edmonton, AB, Canada

## Abstract

Exon skipping using antisense oligonucleotides (ASOs) has recently proven to be a powerful tool for mRNA splicing modulation. Several exon-skipping ASOs have been approved to treat genetic diseases worldwide. However, a significant challenge is the difficulty in selecting an optimal sequence for exon skipping. The efficacy of ASOs is often unpredictable, because of the numerous factors involved in exon skipping. To address this gap, we have developed a computational method using machine-learning algorithms that factors in many parameters as well as experimental data to design highly effective ASOs for exon skipping. eSkip-Finder (https://eskip-finder.org) is the first web-based resource for helping researchers identify effective exon skipping ASOs. eSkip-Finder features two sections: (i) a predictor of the exon skipping efficacy of novel ASOs and (ii) a database of exon skipping ASOs. The predictor facilitates rapid analysis of a given set of exon/intron sequences and ASO lengths to identify effective ASOs for exon skipping based on a machine learning model trained by experimental data. We confirmed that predictions correlated well with in vitro skipping efficacy of sequences that were not included in the training data. The database enables users to search for ASOs using queries such as gene name, species, and exon number.

## Graphical Abstract

**Graphical Abstract:**
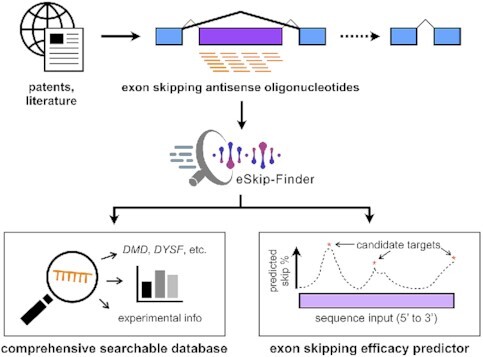
eSkip-Finder uses information on exon skipping antisense oligonucleotides from the literature to produce a database and a skipping efficacy predictive tool to aid researchers in designing effective exon skipping therapies.

## INTRODUCTION

Exon skipping is a strategy that uses antisense oligonucleotides (ASOs) to exclude specific exons from the mature mRNA transcript of a given gene. ASOs are short nucleic acid analogs of diverse chemistry that recognize target mRNA sequences by base pairing. Once hybridized to their targets, ASOs act as steric blockers that prevent splicing factors and other critical proteins from accessing these sequences (1). It is through this mechanism that ASOs could be designed to modulate splicing, for example, by targeting exonic splice enhancer sequences. Given its simplicity and versatility, exon skipping has evolved to become a promising treatment for various genetic disorders, particularly muscular dystrophies (2,3).

Exon skipping is showing promise as a therapy to treat Duchenne muscular dystrophy (DMD) and other genetic diseases (1). Most cases of DMD are caused by large, out-of-frame deletions in the *DMD* gene, leading to an absence of the sarcolemma-stabilizing dystrophin protein in muscle cells (4–6). Exon skipping was adapted to make out-of-frame *DMD* mutations in-frame by removing incompatible exons from the final transcript. In this manner, exon skipping facilitates the production of shorter but partially functional dystrophin protein in muscle, ameliorating DMD pathology. Recent years have seen the approval of four exon-skipping ASOs for DMD therapy by the U.S. Food and Drug Administration (FDA): eteplirsen (2016, Sarepta), golodirsen (2019, Sarepta), viltolarsen (2020, NS and NS Pharma), and casimersen (2021, Sarepta) (7–9). In addition, the FDA approved the first n-of-1 clinical trial with an exon-skipping ASO named milasen to treat a single patient with Batten's disease in 2018 (10).

While these support the outlook of exon skipping as a viable therapeutic strategy for genetic diseases, there is much to improve especially regarding efficacy. For instance, eteplirsen could only restore up to about 1% dystrophin of healthy levels after 180 weeks of treatment in DMD patients (7). Previous studies from our group demonstrate the utility of *in silico* methods to design more effective ASOs (11–14). In one study, we developed an ASO with 12-fold higher *in vitro* exon skipping efficacy than eteplirsen using an *in silico* predictive tool based on statistical modelling (12). Such work and others have since uncovered numerous factors that could influence the exon skipping efficacy of an ASO including length, proximity to splice sites, target mRNA secondary structure, chemistry, and binding energy, among others (13,15–19)—all of which would be useful considerations in ASO design. However, previously developed online tools lack the capacity to simultaneously integrate many parameters critical to ASO design.

**Figure 1.**
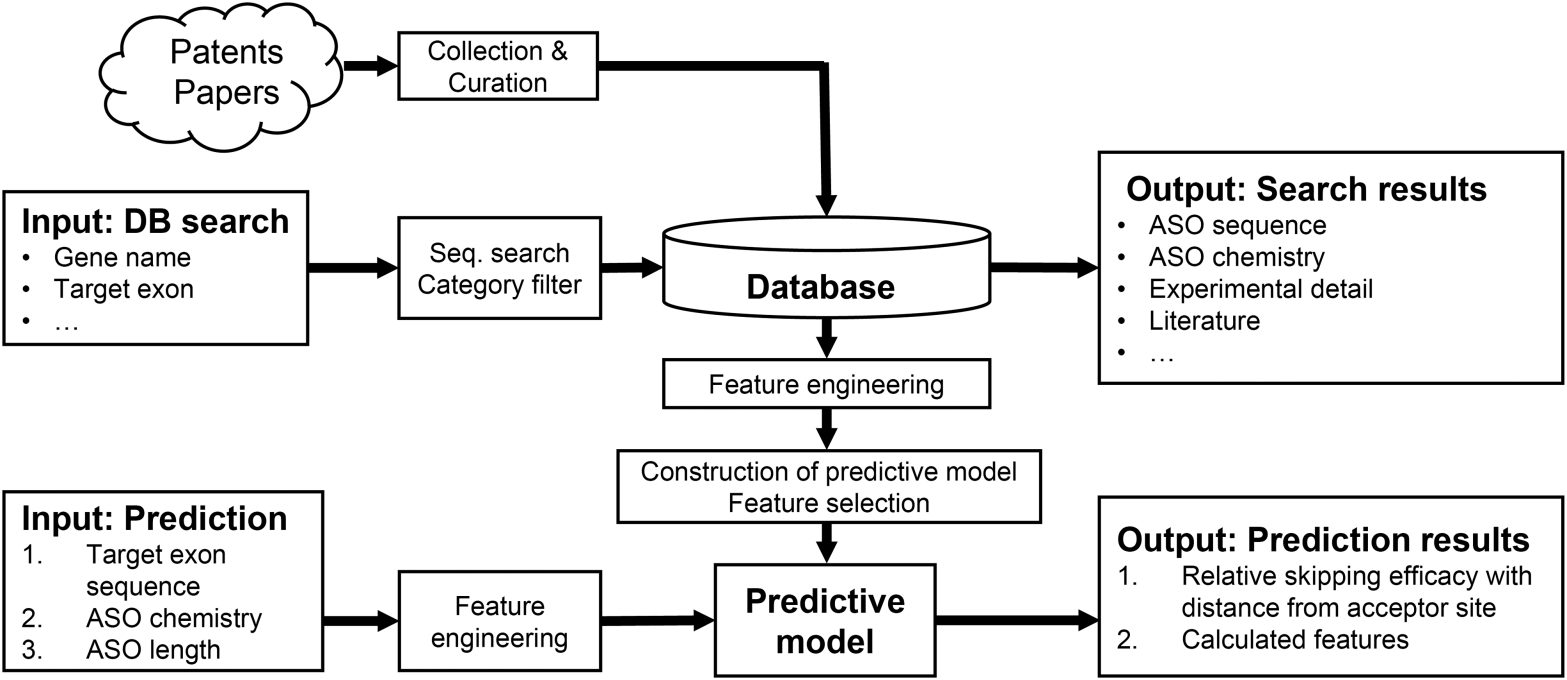
Overview of eSkip-Finder.

To address this gap, we previously developed a computational method using a mathematical model based on 60 descriptor candidates as well as experimental data to design highly effective ASOs for exon skipping (13). Here, we improved this framework further using machine-learning algorithms and have developed eSkip-Finder, a web server to aid the design of effective ASOs for exon skipping. The overview of the webserver is presented in Figure 1. One part of eSkip-Finder is a first-of-its-kind comprehensive database of exon skipping ASOs for *DMD* and other genes. This database was populated using published scientific literature and patents as sources, and contains information such as ASO chemistry, ASO sequence, and experimentally obtained skipping efficacies. The second part is a first-of-its-kind machine learning-based application to predict highly effective ASO sequences for exon skipping, based on a training set of 566 skipping values from 209 unique ASOs extracted from the database above. Here, we describe the features of eSkip-Finder in-depth and outline the ways by which it can be used for the design of exon skipping ASOs.

## RESULTS

### Construction of database

A database of exon-skipping ASOs and their skipping efficacy was built by manually collecting and curating research papers and patents written in English. The database compiles data on exon-skipping ASOs for various genes, including their sequence, target exon, chemistry, literature information, and experimental information such as the ASO concentration, the cell type used for testing, and the target species. The database statistics as of 15 April 2021, are shown in Supplementary Table S1. The complete dataset extracted for each ASO in the database is provided in the web server.

### Predictive model of exon-skipping efficacy

We extracted skipping data that met the following criteria from the database to prepare our training and test datasets: (i) an absolute skipping efficacy was given by a numerical value; (ii) ASO concentration used in the experiment was given; (iii) rhabdomyosarcoma (RD) cells were used in the experiment to normalize experimental conditions; (iv) the skipping efficacy was not given as an EC_50_ value; (v) an ASO sequence that was sequential (not dual-targeting) in the pre-mRNA of dystrophin was used. After filtering the database, 426 skipping values from 109 unique ASO sequences and 228 skipping values from 124 unique ASO sequences were obtained for ASOs with phosphorodiamidate morpholino oligomers (PMO) and 2′-*O*-methyl oligonucleotides (2OMe), respectively. Predictive models were built for the PMO and 2OMe separately. We split the filtered data into a training set (90%) and a test set (10%), as shown in Supplementary Table S2, under two conditions, that is, training and test sets reproduced a similar distribution of skipping efficacy, and they did not share identical sequences, as shown in Supplementary Figure S1.

**Figure 2.**
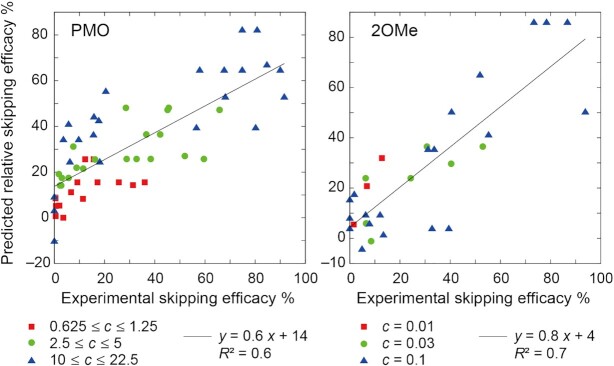
Predictive performance of SVR models for PMO and 2OMe. Symbols represent oligomer concentration (*c*) given in μM used in the experiment. The coefficient of determination, *R*^2^, was calculated by linear regression (black lines).

We built a predictive model for the relative skipping efficacy of a target exon of dystrophin mRNA using the support vector regressor (SVR) implemented in scikit-learn version 0.23.2 (20). First, 32 features, tabulated in Table 1 and Supplementary Table S3, were prepared by feature engineering of ASO and/or its target exon sequences such as predicted binding score between the ASO and its target exon (21), predicted local RNA structure at the target site (22), and GC contents of the ASO and target exon. We also included the ASO concentration used in experimental studies as a feature. More details on the features used are provided elsewhere (13). Each feature was standardized before fitting the model. To select fewer important features, we built all possible combinations of the SVR model that used fewer than seven features, where the experimental ASO concentration was always included as a selected feature. The upper limit number of features, six, was chosen according to the available computational resources. For each model, the hyper-parameter optimization by a grid search for C, gamma, and epsilon was conducted with 100-time repeated splitting of the training data into 80% used to build a model and 20% used to validate the built model under the condition that they did not share identical sequences. Finally, we selected the SVR model that yielded the highest average *R*^2^ of the validation sets as shown in Supplementary Figure S2, the features of which are given in Table 1. The selected models for PMO and 2OMe were applied to the test set, yielding *R*^2^ values of 0.6 and 0.7, as shown in Figure 2. The correlation between experimental and predicted skipping efficacy was confirmed for various concentrations. The contributions of each feature to predictive performance (feature importance) were estimated by permutation importance (23). The importance of each feature was defined by decrease of the *R*^2^ value when the feature in the test set was permutated randomly. The feature importance calculation was repeated 100 times and the averaged values are shown in Table 1. The current model is focused on the prediction of the relative skipping efficacy of ASOs. However, other parameters should be also considered when designing ASOs, one of which is the off-target effect. Other bioinformatics tools such as SKIP-E (https://skip-e.geneticsandbioinformatics.eu/) could complement it.

**Table 1.** Selected features

Selected features for PMO			Selected features for 2OMe		
Name	Description	FI^a^	Name	Description	FI^a^
ASO concentration^b^	Concentration of oligomer used in the experiment	0.64±0.14	ASO concentration^b^	Concentration of oligomer used in the experiment	0.11±0.05
Exon v intron %GC after blocking by oligo	%GC in exon when blocked by oligo / %GC 5′ intron 200 bases upstream	0.68±0.15	GCs (number of)	Total GCs in ASO sequence	0.67±0.20
dG (50BaseFlanksAroundTarget)	Predicted binding energy (21) of ASO to the target sequence plus 50-base flanks (13)	0.66±0.16	ACP	Distance in bases from the splice acceptor site to the center of the target site (17)	0.49±0.21
ACC_LAST15	Predicted accessibility scores (22) of the 3′ end of the target (last 15 bases)	0.32±0.09	%GC of exon when blocked by oligo	Total remaining %GCs of target exon sequence when blocked by ASOs	0.46±0.11
			niscore_per_base	Cumulative NI score (24) divided by the number of exon bases.	0.18±0.09
			ACC_LAST8	Predicted accessibility scores of the 3′ end of the target (last 8 bases)	0.12±0.07

^a^The feature importance (FI) was calculated by the permutation importance method (23).

^b^The ASO concentration used in the experiment is always included as one of the features of the predictive model.

### Implementation

The selected predictive models (Figure 2 and Table 1) are implemented on the web server with scikit-learn (20). Features of local accessibility scores of target exon sequences and binding scores between ASOs and their target exons were calculated with the ViennaRNA Package (22) and RNAstructure (21). The dictionary of NI scores was retrieved from Ref. (24). The concentrations of ASOs were set to typical values, that is, 3 μM for PMO and 0.1 μM for 2OMe. The database was built using PostgreSQL.

### Case study

#### Database search

The web server provides an intuitive search interface of relevant information on exon skipping efficacy with search queries, such as gene name, species, and exon number.

#### Prediction of the efficacy of exon-skipping ASOs

**Figure 3.**
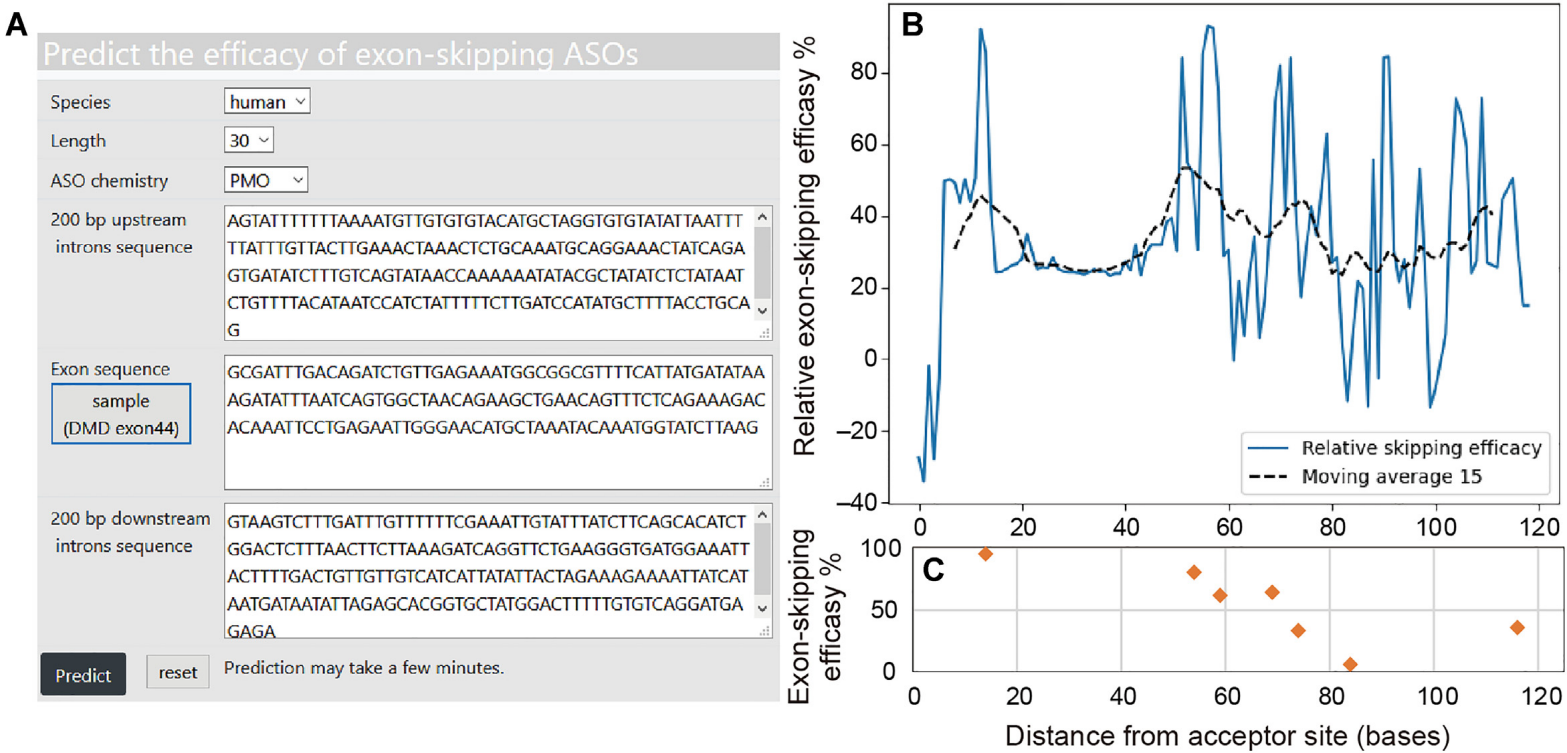
Case study on predicting skipping ASOs for exon 44 of the dystrophin pre-mRNA. (**A**) Input image of the predictive model. A user specifies the length of ASO and its chemistry (PMO or 2OMe). The upstream (200 bases) and downstream (200 bases) intron sequences of the target exon are required in addition to the target exon sequence, which are used to calculate features. (**B**) Output image. The relative exon-skipping efficacy is predicted by scanning the target exon sequence with a window size of the length specified by the user. Moving averages with 15 bases are plotted with a dashed line. (**C**) Efficacy of dystrophin exon 44 skipping observed under identical experimental conditions (cell type used = healthy primary human myotubes, ASO chemistry = PMO, ASO length = 30, ASO concentration = 0.5 μM) as previously reported (15), which is not included in the training dataset. The correlation between predicted and experimental skipping efficacies *R*^2^ was 0.7 as shown in Supplementary Figure S3.

The web server provides a prediction of the relative exon-skipping efficacy of a target exon specified by a user as shown in Figure 3 under the following conditions: 3 μM of PMO or 0.1 μM of 2OMe introduced into cultured cells.

In this case study, we targeted exon 44 of the dystrophin pre-mRNA using a single ASO, the chemistry and length of which were PMO and 30-mer, respectively. A user needs to input 200 bp of upstream and 200 bp of downstream intron sequences in addition to the target exon sequence, as this sequence information is required to calculate the features. The prediction took 79 s. We obtained the promising regions for exon 44 skipping, that is, the regions between 10–20 and 50–80. We found that these regions were indeed included in experimentally observed effective ASOs (15).

As a validation of predicting exon skipping efficiency beyond *DMD*, we present a test case of PMO-mediated exon 73 skipping of collagen type VII alpha 1 chain (*COL7A1*) (Supplementary Table S4). (25). Although the experimental conditions (e.g. ASO concentration) were different, we found that predicted and experimental values correlated well with each other, and the model correctly ranked the efficacy of the three PMOs, indicating a potential predictive ability of the current model for other genes. Currently, the amount of available experimental data of exon skipping for other genes is limited. To examine the applicability of our model to other genes, we plan to further validate with various genes when sufficient data become available. We expect that adding various genes and oligo chemistries to the database will help expand the applicability of the predictive model further.

## DATA AVAILABILITY

The authors confirm that the data supporting the findings of this study are available within the article and/or in the supplementary material.

## Supplementary Material

gkab442_Supplemental_File

## References

[1] Lim K.R.Q. , Yokota T. Invention and early history of exon skipping and splice modulation. Methods Mol. Biol. 2018; 1828:3–30. 30171532 10.1007/978-1-4939-8651-4_1

[2] Rodrigues M. , Yokota T. Yokota T. , Maruyama R. Exon Skipping and Inclusion Therapies: Methods and Protocols. 2018; NY Springer 31–55.

[3] Siva K. , Covello G., Denti M.A. Exon-skipping antisense oligonucleotides to correct missplicing in neurogenetic diseases. Nucleic Acid Ther. 2014; 24:69–86. 24506781 10.1089/nat.2013.0461 PMC3922311

[4] Petrof B.J. , Shrager J.B., Stedman H.H., Kelly A.M., Sweeney H.L. Dystrophin protects the sarcolemma from stresses developed during muscle contraction. Proc. Natl. Acad. Sci. U.S.A. 1993; 90:3710–3714. 8475120 10.1073/pnas.90.8.3710 PMC46371

[5] Bladen C.L. , Salgado D., Monges S., Foncuberta M.E., Kekou K., Kosma K., Dawkins H., Lamont L., Roy A.J., Chamova T. et al. The TREAT-NMD DMD global database: analysis of more than 7,000 Duchenne muscular dystrophy mutations. Hum. Mutat. 2015; 36:395–402. 25604253 10.1002/humu.22758 PMC4405042

[6] Tuffery-Giraud S. , Béroud C., Leturcq F., Yaou R.B., Hamroun D., Michel-Calemard L., Moizard M.-P., Bernard R., Cossée M., Boisseau P. et al. Genotype–phenotype analysis in 2,405 patients with a dystrophinopathy using the UMD–DMD database: a model of nationwide knowledgebase. Hum. Mutat. 2009; 30:934–945. 19367636 10.1002/humu.20976

[7] Lim K.R. , Maruyama R., Yokota T. Eteplirsen in the treatment of Duchenne muscular dystrophy. Drug Des. Dev. Ther. 2017; 11:533–545. 10.2147/DDDT.S97635 PMC5338848 28280301

[8] Anwar S. , Yokota T. Golodirsen for Duchenne muscular dystrophy. Drugs Today (Barc.). 2020; 56:491–504. 33025945 10.1358/dot.2020.56.8.3159186

[9] Roshmi R.R. , Yokota T. Viltolarsen for the treatment of Duchenne muscular dystrophy. Drugs Today (Barc.). 2019; 55:627–639. 31720560 10.1358/dot.2019.55.10.3045038

[10] Kim J. , Hu C., Moufawad El Achkar C., Black L.E., Douville J., Larson A., Pendergast M.K., Goldkind S.F., Lee E.A., Kuniholm A. et al. Patient-customized oligonucleotide therapy for a rare genetic disease. N. Engl. J. Med. 2019; 381:1644–1652. 31597037 10.1056/NEJMoa1813279 PMC6961983

[11] Echigoya Y. , Lim K.R.Q., Melo D., Bao B., Trieu N., Mizobe Y., Maruyama R., Mamchaoui K., Tanihata J., Aoki Y. et al. Exons 45–55 skipping using mutation-tailored cocktails of antisense morpholinos in the DMD gene. Mol. Ther. 2019; 27:2005–2017. 31416775 10.1016/j.ymthe.2019.07.012 PMC6838919

[12] Echigoya Y. , Lim K.R.Q., Trieu N., Bao B., Miskew Nichols B., Vila M.C., Novak J.S., Hara Y., Lee J., Touznik A. et al. Quantitative antisense screening and optimization for Exon 51 skipping in Duchenne muscular dystrophy. Mol. Ther. 2017; 25:2561–2572. 28865998 10.1016/j.ymthe.2017.07.014 PMC5675502

[13] Echigoya Y. , Mouly V., Garcia L., Yokota T., Duddy W. In silico screening based on predictive algorithms as a design tool for exon skipping oligonucleotides in Duchenne muscular dystrophy. PLoS One. 2015; 10:e0120058. 25816009 10.1371/journal.pone.0120058 PMC4376395

[14] Lee J.J.A. , Maruyama R., Duddy W., Sakurai H., Yokota T. Identification of novel antisense-mediated exon skipping targets in DYSF for therapeutic treatment of dysferlinopathy. Mol. Ther. - Nucleic Acids. 2018; 13:596–604. 30439648 10.1016/j.omtn.2018.10.004 PMC6234522

[15] Popplewell L.J. , Trollet C., Dickson G., Graham I.R. Design of phosphorodiamidate morpholino oligomers (PMOs) for the induction of exon skipping of the human DMD gene. Mol. Ther. 2009; 17:554–561. 19142179 10.1038/mt.2008.287 PMC2835085

[16] Harding P.L. , Fall A.M., Honeyman K., Fletcher S., Wilton S.D. The influence of antisense oligonucleotide length on dystrophin exon skipping. Mol. Ther. 2007; 15:157–166. 17164787 10.1038/sj.mt.6300006

[17] Pramono Z.A.D. , Wee K.B., Wang J.L., Chen Y.J., Xiong Q.B., Lai P.S., Yee W.C. A prospective study in the rational design of efficient antisense oligonucleotides for exon skipping in the DMD gene. Hum. Gene Ther. 2012; 23:781–790. 22486275 10.1089/hum.2011.205 PMC3404420

[18] Wee K.B. , Pramono Z.A.D., Wang J.L., MacDorman K.F., Lai P.S., Yee W.C. Dynamics of co-transcriptional pre-mRNA folding influences the induction of dystrophin exon skipping by antisense oligonucleotides. PLoS One. 2008; 3:e1844. 18365002 10.1371/journal.pone.0001844 PMC2267000

[19] Aartsma-Rus A. , Houlleberghs H., van Deutekom J.C.T., van Ommen G.-J.B., t Hoen P.A.C. Exonic sequences provide better targets for antisense oligonucleotides than splice site sequences in the modulation of Duchenne muscular dystrophy splicing. Oligonucleotides. 2010; 20:69–77. 20377429 10.1089/oli.2009.0215

[20] Pedregosa F. , Varoquaux G.e., Gramfort A., Michel V., Thirion B., Grisel O., Blondel M., Prettenhofer P., Weiss R., Dubourg V. et al. Scikit-learn: machine learning in Python. J. Mach. Learn. Res. 2011; 12:2825–2830.

[21] Reuter J.S. , Mathews D.H. RNAstructure: software for RNA secondary structure prediction and analysis. BMC Bioinformatics. 2010; 11:129. 20230624 10.1186/1471-2105-11-129 PMC2984261

[22] Lorenz R. , Bernhart S.H., Höner zu Siederdissen C., Tafer H., Flamm C., Stadler P.F., Hofacker I.L. ViennaRNA Package 2.0. Algorith. Mol. Biol. 2011; 6:26. 10.1186/1748-7188-6-26 PMC3319429 22115189

[23] Altmann A. , Tolosi L., Sander O., Lengauer T. Permutation importance: a corrected feature importance measure. Bioinformatics. 2010; 26:1340–1347. 20385727 10.1093/bioinformatics/btq134

[24] Stadler M.B. , Shomron N., Yeo G.W., Schneider A., Xiao X., Burge C.B. Inference of splicing regulatory activities by sequence neighborhood analysis. PLoS Genet. 2006; 2:e191. 17121466 10.1371/journal.pgen.0020191 PMC1657047

[25] Ham K.A. , Aung-Htut M.T., Fletcher S., Wilton S.D. Nonsequential splicing events alter antisense-mediated exon skipping outcome in COL7A1. Int. J. Mol. Sci. 2020; 21:7705. 10.3390/ijms21207705 PMC7590164 33081018

