# Supplementary material for: eSkip-Finder: a machine learning-based web application and database to identify the optimal sequences of antisense oligonucleotides for exon skipping
Source: Nucleic Acids Res. 2021 Jun 9;49(W1):W193–8. doi: 10.1093/nar/gkab442 (PMC8265194; doi:10.1093/nar/gkab442)
Supplement: gkab442_Supplemental_File [file gkab442_supplemental_file.docx]

Supplemental information for

**eSkip-Finder: a machine learning-based web application and database to identify the optimal sequences of antisense oligonucleotides for exon skipping**

Shuntaro Chiba^1^, Kenji Rowel Q. Lim^2^, Narin Sheri^2^, Saeed Anwar^2^, Esra Erkut^2^, Md Nur Ahad Shah^2^, Tejal Aslesh^2^, Stanley Woo^2^, Omar Sheikh^2^, Rika Maruyama^2^, Hiroaki Takano^1^, Katsuhiko Kunitake^3^, William Duddy^4^, Yasushi Okuno^1,5,*^, Yoshitsugu Aoki^3,*^, and Toshifumi Yokota^2,3,*^

^1^ HPC- and AI-driven Drug Development Platform Division, RIKEN Center for Computational Science, Yokohama, 230-0045, Japan

^2^ Department of Medical Genetics, University of Alberta Faculty of Medicine and Dentistry, 8613-114 St, Edmonton, AB, Canada

^3^ Department of Molecular Therapy, National Institute of Neuroscience, National Center of Neurology and Psychiatry (NCNP), Kodaira, Tokyo 187-8551, Japan

^4^ Northern Ireland Center for Stratified Medicine, Biomedical Sciences Research Institute, C-TRIC, Altnagelvin Hospital Campus, Ulster University, Londonderry BT47 6SB, UK

^5^ Department of Biomedical Data Intelligence, Graduate School of Medicine, Kyoto University, Kyoto, 606-8507, Japan

* To whom correspondence should be addressed. Tel: +1-780-492-1102; Fax: +1-780-492-1998; Email: toshifumi.yokota@ualberta.ca

* Correspondence may also be addressed. Tel: +81-42-346-1720; Fax: +81-42-346-1750; Email: tsugu56@ncnp.go.jp

* Correspondence may also be addressed. Tel: +81-75-751-3920; Fax: +81-75-751-3920; Email: okuno.yasushi.4c@kyoto-u.ac.jp

Table S1. Statistics of the eSkip-Finder database as of April 15, 2021.

| Available genes | | | | | |
| --- | --- | --- | --- | --- | --- |
| *DMD, MSTN, DYSF, SCN1, COL7A1, LAMA2, MAPT, USH2A, DMPK, MS4A2, ATM, ALK2/ACVR1, NF1, PMM2, NPC1, NF2, MLC1, MFSD8* | | | | | |
| # of skipping data (# of unique sequence of ASOs) | | | | | |
| *DMD* | *MSTN* | *DYSF* | *SCN1* | *COL7A1* | *LAMA2* |
| 5137 (1809) | 674 (230) | 69 (35) | 57 (48) | 42 (15) | 23 (23) |
| *MAPT* | *USH2A* | *DMPK* | *MS4A2* | *ATM* | *ALK2/ACVR1* |
| 18 (13) | 7 (1) | 7 (7) | 4 (2) | 4 (4) | 4 (1) |
| *NF1* | *PMM2* | *NPC1* | *NF2* | *MLC1* | *MFSD8* |
| 3 (3) | 1 (1) | 1 (1) | 1 (1) | 1 (1) | 1 (1) |

Table S2. Training and test sets used to build and validate predictive models.

|  | Phosphorodiamidate morpholino oligomers (PMO) | | 2'-*O*-Methyl oligonucleotide (2OMe) | |
| --- | --- | --- | --- | --- |
|  | Training | Test | Training | Test |
| # of skipping efficacy values | 369 | 57 | 197 | 31 |
| # of unique sequences | 98 | 11 | 111 | 13 |
| Exon number and their count in the dataset in parentheses | 2 (13), 44 (98), 45 (67), 50 (36), 51 (4), 53 (121), 55 (30) | 2 (3), 44 (28), 45 (3), 53 (8), 55 (15) | 44 (55), 45 (38), 50 (3), 53 (88), 55 (13) | 44 (2), 45 (5), 53 (11), 55 (13) |

Table S3. Feature candidates not selected in the final predictive models.

| Name | Description |
| --- | --- |
| Distance from acceptor | Position of last base of ASO relative to acceptor site |
| Distance from donor | Position of 1st base of ASO relative to donor site |
| Length | Total bases in ASO |
| Exon Length | Total bases in target exon |
| Exon Malueka Category*^a^* | The Malueka category (1) of target exon |
| Niscore | Cumulative NI score of target sequence (2) |
| ACC_FL | Predicted accessibility score of target sequence (3) |
| ACC_BEST8 | The maximum accessibility score within target sequence (the 8 contiguous bases of the target having the greatest summed accessibility scores) (3) |
| ACC_AVE | Average of all accessibility scores of target sequence (3) |
| %GC oligo | %GC in ASO |
| %GC 5' intron 200 bases upstream | %GC in intron 200 bases upstream of target exon |
| %GC fold increase target over 5' intron | (Total GCs in target sequence / Target length)  / %GC 5' intron 200 bases upstream |
| %GC increase target over 5' intron | (Total GCs in target sequence / Target length)  − %GC 5' intron 200 bases upstream |
| Decrease in GC% due to blocking by oligo | %GC exon  − %GC exon when blocked by oligo |
| Decrease in ratio of exon to intron %GC due to oligo blocking | (%GC exon − %GC exon when blocked by oligo)  / %GC 5' intron 200 bases upstream |
| %GC exon | %GC in target exon |
| Total GCs in exon | Total GCs in target exon |
| # exon GCs blocked by oligo | Total GCs blocked by ASO |
| Length of exon when blocked by oligo | Length of target exon − Length of ASO |
| Exon v intron %GC | %GC exon / %GC 5' intron 200 bases upstream |
| dG (TargetAsExon) | Predicted binding energy of ASO to the target (4) |
| dG (100BaseFlanks) | Predicted binding energy of ASO to target sequence plus 100-base flanks (4) (5) |
| dG (200BaseFlanks) | Predicted binding energy of ASO to target sequence plus 200-base flanks (4) (5) |
| dG (ExonStartTo10BaseDownFromOligo) | Predicted binding energy of ASO to target sequence plus 10 downstream bases (4) (5) |

*a*. This category feature was converted to one-hot vectors.

Table S4. Prediction of exon 73 skipping of collagen type VII alpha 1 chain (COL7A1) using PMOs.

| Input sequence | | | |
| --- | --- | --- | --- |
| Exon 73:  GGCCCCATCGGCTTTCCTGGAGAACGCGGGCTGAAGGGCGACCGTGGAGACCCTGGCCCTCAGGGGCCACCTGGTCTGGCCCTTGGGGAGAGGGGCCCCCCCGGGCCTTCCGGCCTTGCCGGGGAGCCTGGAAAGCCTGGTATTCCCGGGCTCCCAGGCAGGGCTGGGGGTGTGGGAGAGGCAGGAAGGCCAGGAGAGAGG  Upstream 200:  CTGCGGGAGATCGTGGAGACCTGGGATGAGAGCTCTGGTAGCTTCCTGCCTGTGCCCGAACGGCGTCGAGGCCCCAAGGGGGACTCAGGCGAACAGGGCCCCCCAGGCAAGGAGGTGAGCAGAAGTGGCTCAGTGGGTTGTGCCCCGTGGAGTGGGGTGTAGCTGTACAGCCACCAGCATTCTCTCTTCCACTCCTGCAG  Downstream 200:  GTGAGGCTGGGGGCTGGCCAGGAGAGTGAGGGAAGAGGGGTTGGGAGGGGTGGGACCCCCCATGGGCTTGGCCCTCACCCGCTATTTGCATTTCAGGGAGAACGGGGAGAGAAAGGAGAACGTGGAGAACAGGTGGGCTGCGATGGGCTTCGTGGGGCAGGCTGTCTGGAGGCTGTGCTGGGGCTGCCACCCCATTTTCT | | | |
| ASO Name | ASO sequence | Predicted value | Experimental value (6) |
| H73A(+16+40) | CGCCCUUCAGCCCGCGUUCUCCAGG (25 mer) | 60% (ranked #1) | 100% (ranked #1) |
| H73A(+16+35) | UUCAGCCCGCGUUCUCCAGG  (20 mer) | 23% (ranked #3) | 40% (ranked #3) |
| H73A(+21+40) | CGCCCUUCAGCCCGCGUUCU  (20 mer) | 48% (ranked #2) | 85% (ranked #2) |


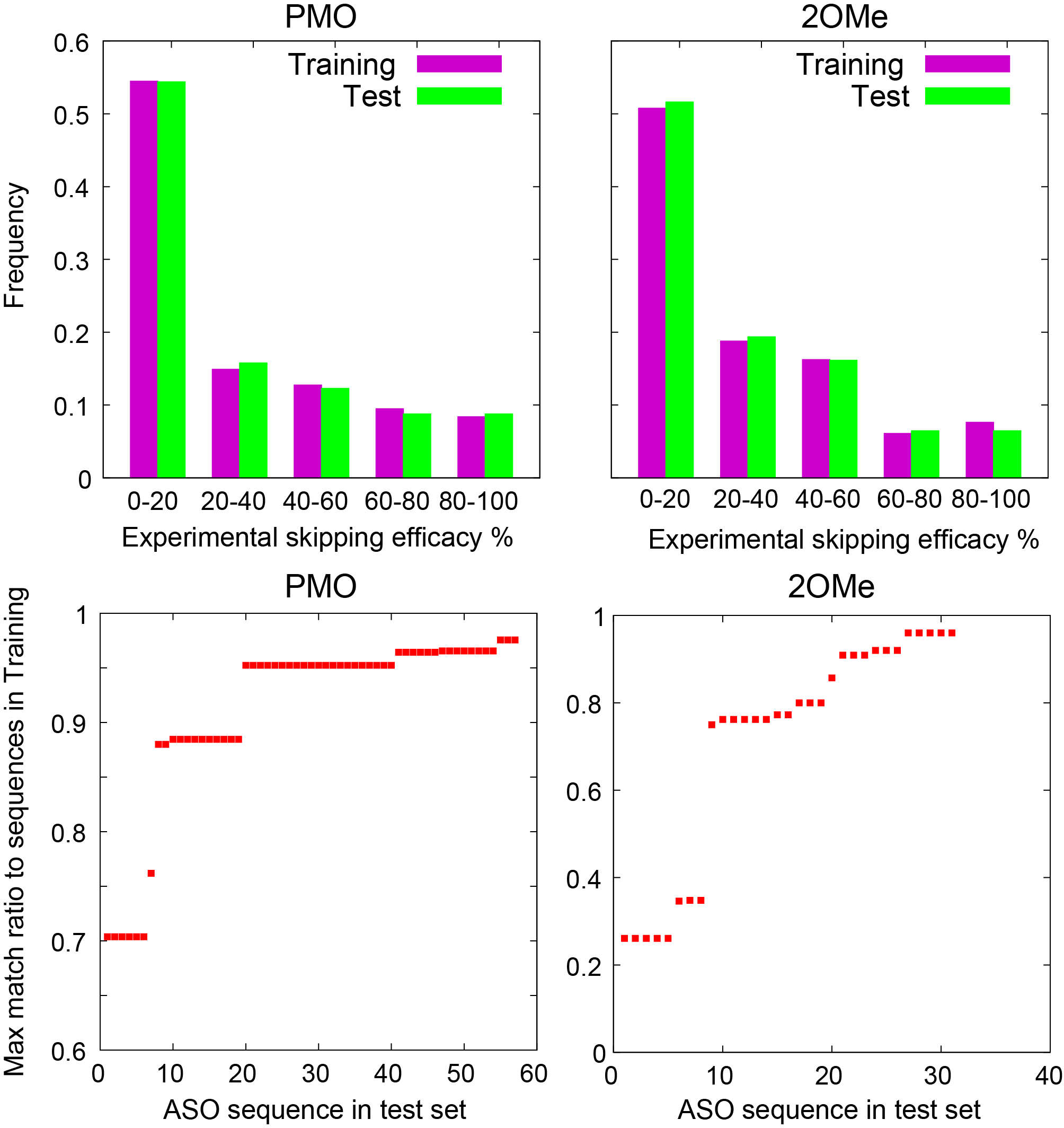


Figure S1. (Upper) Histogram of skipping efficacy in training and test sets. (Lower) Each ASO sequence in the test set was compared to all the sequences in the training set. The match ratio defined as 2 × # matched sequence / (# of test sequence + # of training sequence) was calculated. The highest match ratio is plotted.


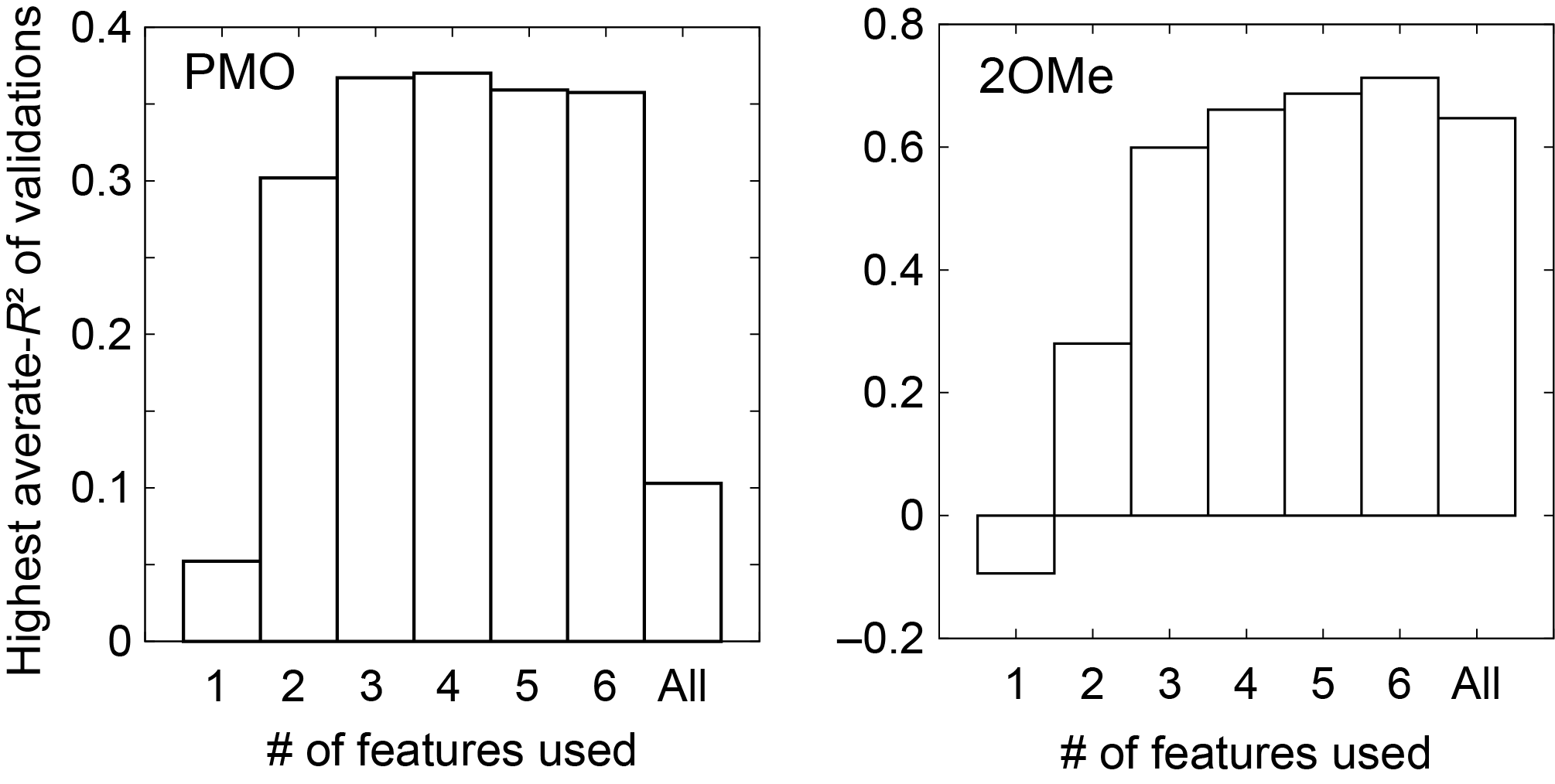


Figure S2. Feature selection of SVR models for PMO and 2OMe. The experimental ASO concentration was always included as a selected feature. For example, when # of features used equals 6, all SVR models that used five features out of 32 features were built and evaluated.


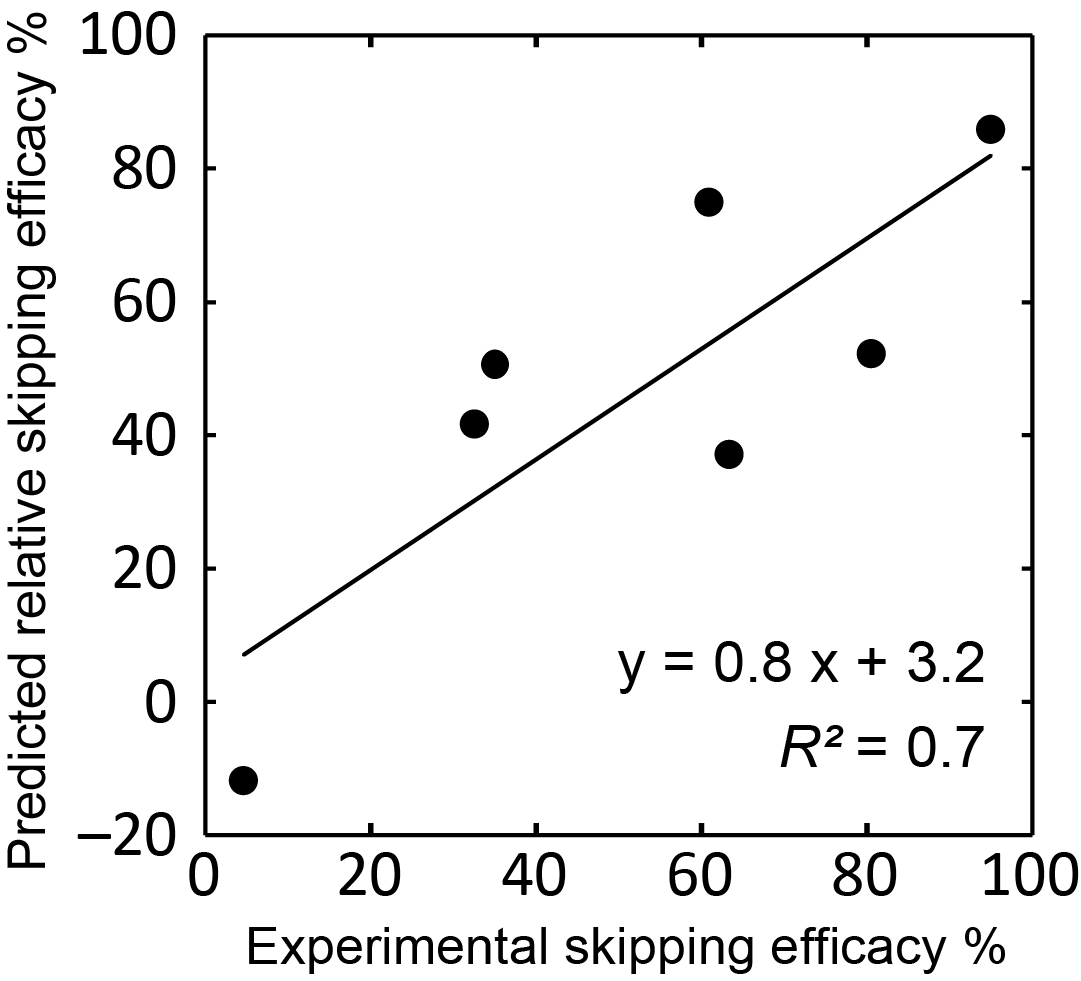


Figure S3. Comparison of experimental (7) and predicted relative skipping efficacies of exon 44 of *DMD* shown in Figure 3 as a test case.

**References**

1. Malueka, R.G., Takaoka, Y., Yagi, M., Awano, H., Lee, T., Dwianingsih, E.K., Nishida, A., Takeshima, Y. and Matsuo, M. (2012) Categorization of 77 dystrophin exons into 5 groups by a decision tree using indexes of splicing regulatory factors as decision markers. *BMC Genet*, **13**, 23, doi:10.1186/1471-2156-13-23.

2. Stadler, M.B., Shomron, N., Yeo, G.W., Schneider, A., Xiao, X. and Burge, C.B. (2006) Inference of Splicing Regulatory Activities by Sequence Neighborhood Analysis. *PLOS Genetics*, **2**, e191, doi:10.1371/journal.pgen.0020191.

3. Lorenz, R., Bernhart, S.H., Höner zu Siederdissen, C., Tafer, H., Flamm, C., Stadler, P.F. and Hofacker, I.L. (2011) ViennaRNA Package 2.0. *Algorithms for Molecular Biology*, **6**, 26, doi:10.1186/1748-7188-6-26.

4. Reuter, J.S. and Mathews, D.H. (2010) RNAstructure: software for RNA secondary structure prediction and analysis. *BMC Bioinformatics*, **11**, 129, doi:10.1186/1471-2105-11-129.

5. Echigoya, Y., Mouly, V., Garcia, L., Yokota, T. and Duddy, W. (2015) In silico screening based on predictive algorithms as a design tool for exon skipping oligonucleotides in Duchenne muscular dystrophy. *Plos One*, **10**, e0120058, doi:10.1371/journal.pone.0120058.

6. Ham, K.A., Aung-Htut, M.T., Fletcher, S. and Wilton, S.D. (2020) Nonsequential Splicing Events Alter Antisense-Mediated Exon Skipping Outcome in COL7A1. *Int J Mol Sci*, **21**, doi:10.3390/ijms21207705.

7. Popplewell, L.J., Trollet, C., Dickson, G. and Graham, I.R. (2009) Design of Phosphorodiamidate Morpholino Oligomers (PMOs) for the Induction of Exon Skipping of the Human DMD Gene. *Molecular Therapy*, **17**, 554-561, doi:10.1038/mt.2008.287.
